# The impact of generative AI use on employees’ psychological distress: a moderated mediation model

**DOI:** 10.3389/fpubh.2026.1798423

**Published:** 2026-05-20

**Authors:** Meng Liu, Yang Li, Jing Li

**Affiliations:** 1 School of Management, Tianjin University of Commerce, Tianjin, China; 2 Research Center for Management Innovation and Evaluation, Tianjin University of Commerce, Tianjin, China

**Keywords:** AI ethical risk perception, Gen AI, information literacy, job insecurity, psychological distress, workplace loneliness

## Abstract

**Introduction:**

With the widespread adoption of generative artificial intelligence (Gen AI) in the workplace, concerns have emerged about whether employees experience psychological distress alongside efficiency improvements.

**Methods:**

Based on the Stimulus-Organism-Response (SOR) framework, we developed a moderated mediation model and used survey data from 424 Chinese employees to examine the relationship between Gen AI use and employees’ psychological distress. We tested dual mediating pathways (job insecurity and workplace loneliness) and two boundary conditions (information literacy and AI ethical risk perception).

**Results:**

Our study reveals that Gen AI use is significantly associated with psychological distress via the dual mediating paths of job insecurity and workplace loneliness. Employees’ information literacy can mitigate the relationship between Gen AI and job insecurity, and further moderate the mediating effect on psychological distress. Conversely, AI ethical risk perception strengthens the relationship between Gen AI and workplace loneliness, and is further associated with psychological distress via a moderated mediation effect.

**Discussion:**

Our study contributes to the theoretical understanding and empirical evidence regarding the relationship between Gen AI and employees’ psychological distress. It also provides practical recommendations for managers and employees on how to interact effectively with Gen AI tools.

## 1 Introduction

Generative artificial intelligence (Gen AI) has become a pivotal tool for enhancing efficiency, optimizing decision-making, and driving innovation by extracting vast amounts of information from data and using advanced machine learning algorithms to create human-like content outputs (1, 2). Gen AI tools serve as virtual partners, enabling employees to enhance productivity by interacting with them to solve work-related problems. However, alongside these advantages, emerging surveys suggest that the widespread adoption of Gen AI has introduced substantial psychological challenges for employees. For instance, a recent survey by the Pew Research Center found that 52% of workers are worried about the impact of AI on their jobs and 33% report feeling overwhelmed by AI-related changes (3). Consistent with these findings, survey data from Tencent Research Institute indicate that AI adoption in China also generates dual career-related concerns: 77% report at least moderate anxiety that their professional skills may become obsolete, and 70% worry about potential job displacement (4). This evidence highlights a growing psychological burden associated with Gen AI use, demonstrating the urgency of examining its impact on employees’ occupational health and workplace outcomes. Building on these emerging real-world concerns, recent studies have also focused on the negative consequences of AI use, such as causing job insecurity (5), increasing turnover intention (6), damaging psychological well-being (7), and increasing work–family conflict (8).

Despite its significance, the current understanding of the negative impacts of Gen AI use remains limited. To our knowledge, systematic research has yet to examine the relationship between Gen AI use and employees’ psychological distress. Psychological distress manifests as anxiety, depression, stress, etc. (9), leading to diminished confidence in job responsibilities, reduced work efficiency (10, 11), and increased risk of various behavioral disorders and illnesses (12). Although recent studies have found that (Gen) AI increases fatigue (13), negative emotions (14), emotional exhaustion (15), and psychological stress (16), the evidence remains fragmented because measures of psychological distress encompass multiple dimensions. Except for the study by Zheng and Zhang (15), the current literature has yet to fully elucidate the underlying mechanisms. Specifically, whether employees experience psychological distress as an unintended consequence of using Gen AI for efficiency remains an open question. This question, along with the mechanisms by which Gen AI is associated with psychological distress, need to be clarified.

In addition, it is necessary to identify the boundary conditions between Gen AI use and adverse impact outcomes. First, some studies have attempted to identify factors that mitigate this relationship, such as organizational support (17), retraining (5), trait resilience (8, 18), and conscientiousness (19). However, these variables either require organizational intervention or are inherent individual traits that are difficult to change. Therefore, it is crucial to identify individual-level competencies that enable employees to effectively manage and mitigate the potential adverse effects of using Gen AI. In contrast, the current literature provides limited insight into the factors that exacerbate the relationship between Gen AI use and adverse outcomes. Among these factors, Stahl and Eke argue that AI ethical issues related to Gen AI are essential considerations, such as a lack of accountability, privacy violations, unfairness, and opacity (20). However, Bai et al. (21) found that few studies have examined the ethical aspects of Gen AI use to provide a more balanced view of its usage impact. Current literature still focuses primarily on whether ethical perception is associated with individuals’ intentions to use Gen AI (22, 23). In reality, as Gen AI proliferates across various industries (24), people are increasingly likely to use Gen AI while grappling with ethical dilemmas. Furthermore, there is a lack of literature verifying whether AI ethics exacerbates the relationship between Gen AI use and psychological distress.

To address these gaps, this study aims to construct and test a mechanistic framework linking Gen AI use to employees’ psychological distress. Drawing on Stimulus-Organism-Response (SOR) theory, we develop a moderated mediation model in which Gen AI use serves as the stimulus, job insecurity and workplace loneliness as dual organismic mediators, and psychological distress represents the response outcome. Furthermore, information literacy and AI ethical risk perception are tested as potential boundary conditions. Using survey data collected from 424 employees in China and structural equation modeling, we test the proposed relationships in an integrated framework.

Our study makes several primary contributions to the literature on psychological distress. First, guided by the SOR model, our study advances understanding of the relationship between Gen AI use and employees’ psychological distress by highlighting the roles of job insecurity and workplace loneliness as key mediating pathways, thereby filling the gap in the literature concerning the underlying mechanisms. Second, we identify information literacy as a boundary condition that shapes the relationship between Gen AI use and employee outcomes, offering a novel perspective on how employees may alleviate job insecurity and psychological distress. Third, to our knowledge, we provide the first empirical evidence that AI ethical risk perception serves as an exacerbating boundary condition along the workplace loneliness pathway, thereby supplementing the literature on the ethical implications of Gen AI use.

## 2 Theory and hypotheses development

### 2.1 Stimulus-organism-response (SOR) theory

The SOR theoretical framework emphasizes that responses and behavioral outcomes are not direct products of external stimuli but rather reactions formed through the processing of individuals’ emotional and cognitive states triggered by environmental stimuli (25). While originally applied to environmental psychology and consumer behavior research (26, 27), SOR theory has been adapted and validated in the contexts of technological change (26, 28). Studies have confirmed that the SOR model can effectively explain how technology-induced cognitive threats, uncertainty, loss of control, and information overload are related to employees’ anxiety, fatigue, and psychological exhaustion (28–30). For instance, Duong et al. (28) developed a model of psychological adaptation mechanisms based on the SOR framework to explain that the extensive use of ChatGPT contributes to increased anxiety, burnout, and sleep disturbances among employees. These findings suggest that the SOR model can be applied to technological contexts and demonstrate strong theoretical adaptability in explaining how external stimuli are associated with individual responses.

**Figure 1:**
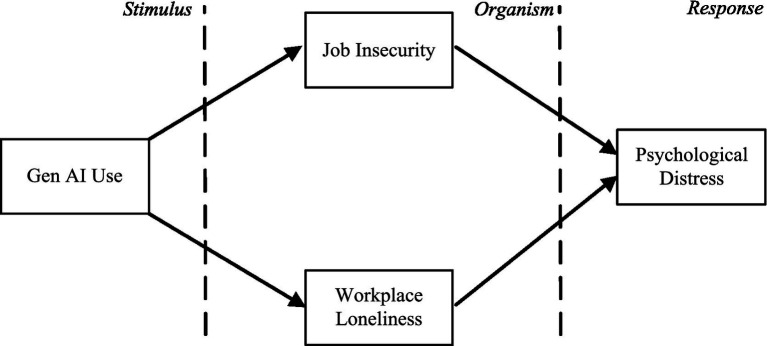
Baseline analytical framework based on SOR theory.

Based on SOR theory, we explore the mechanism through which Gen AI influences employees’ psychological distress. First, as Gen AI permeates the organizational environment, its integration acts as a significant environmental stimulus (S). Consequently, employees may exhibit organismic perception (O) at both the cognitive and emotional levels. On the one hand, employees may experience insecurity at the cognitive level, such as skill devaluation and role replacement (31). On the other hand, the reduction or replacement of interpersonal collaboration and communication due to Gen AI use may trigger workplace loneliness among employees at the emotional level (32). Ultimately, the external stimulus (S) propagates through these organismic perceptions (O) to shape the psychological response (R). The resultant manifestations include persistent psychological distress (e.g., anxiety, emotional exhaustion) and may even escalate to physiological symptoms like sleep disturbances or physical ailments (28, 32). As shown in Figure 1, we construct a baseline analytical framework based on SOR theory to examine how Gen AI use is associated with employees’ psychological distress.

### 2.2 Hypotheses development

#### 2.2.1 The stimulus of gen AI use (S) on job insecurity and workplace loneliness (O)

Job insecurity is defined as employees’ overall concern about the continuity and stability of their own jobs. The rapid advancement of Gen AI imposes a compelling stimulus on employees and may trigger organic reactions such as job insecurity (15, 28). Prior research suggests that the core driver of individual behavior is the ability to maintain goal states, which is achieved through continuous feedback and behavioral adjustment. When individuals perceive that their behaviors fail to yield expected feedback, their sense of control weakens (33, 34). Within the context of employment, this sense of control is predominantly manifested in employees’ proficiency in their roles, career progression, and job stability (35). Gen AI serves as an environmental stimulus that disrupts this feedback mechanism. The pervasive implementation of Gen AI is precipitating a paradigm shift in the established relationship between employees and their professional objectives, engendering a phenomenon of “loss of control.” This phenomenon manifests in two distinct dimensions: First, a failure in skill feedback occurs. The advent of Gen AI has reshaped the nature of employment, as tasks previously mastered by employees are replaced by automated processes. This has engendered a sense of professional inadequacy among employees, who perceive a diminution in the value of their skills. Consequently, employees are compelled to question the relevance of their personal efforts in achieving career advancement or stability (36). Second, role feedback becomes ambiguous. Gen AI reshapes task structures and job boundaries, resulting in blurred roles and constantly changing responsibilities. This reduces employees’ sense of control over their own role positioning and career paths (31, 32). Job insecurity is the subjective reflection of this “control imbalance,” essentially stemming from employees’ subjective doubts about the controllability of their future careers (35). The technological threats and role uncertainty associated with Gen AI may drive employees to evaluate their job security. Our study proposes the following hypothesis:

*H1a:* Gen AI use is positively associated with employees' job insecurity.

Workplace loneliness is defined as a negative emotion resulting from dissatisfaction with workplace relationships (37). In the SOR framework, workplace loneliness also represents an affective organismic state triggered by environmental changes in social interaction patterns (38, 39). The Social Isolation Model posits that as social beings, humans rely on “sustained, effective, and meaningful social connections” for psychological stability and emotional fulfillment. These connections must meet three core needs—emotional resonance, instrumental support, and role identity—rather than merely accumulating frequent social interactions (40, 41). When such social connections are disrupted or weakened, individuals experience objective isolation and subjective loneliness (39). The widespread adoption of Gen AI undermines the stability of these social connections in two ways, thereby shaping their emotional perception of workplace loneliness. First, while AI improves task efficiency, it replaces natural work-based interactions (e.g., face-to-face discussions) (32). Particularly in remote work and automated workflow environments, employees struggle to access key social cues such as nonverbal empathy and personalized support. Consequently, they cannot maintain their sense of belonging through interactions, and the lack of social feedback gradually gives rise to loneliness (31, 32). Second, with the development of “emotional substitution,” employees’ emotional needs may be temporarily satisfied through interactions with AI. However, AI interactions cannot replace real interpersonal connections. Immersion in “virtual emotions” may trigger “authenticity anxiety,” further exacerbating employees’ loneliness (42). Therefore, the study proposes the following hypothesis:

*H1b:* Gen AI use is positively associated with employees' workplace loneliness.

#### 2.2.2 The direct and mediating roles of job insecurity (O) on psychological distress (R)

The individuals’ internal cognitive evaluations or emotional states further drive them to respond positively or negatively (27). Job insecurity disrupts employees’ psychological balance, leaving them in a state of long-term uncertainty about their career future and thereby laying the groundwork for negative psychological distress (35). Existing studies have shown that job insecurity is closely correlated with psychological distress states such as anxiety, depression, and emotional exhaustion (35, 43, 44). Employees who perceive job insecurity over the long-term exhibit significantly lower levels of psychological well-being, more depressive symptoms, and poorer overall health compared to those with stable employment. This indicates that such uncertainty acts as a chronic stressor, continuously depleting employees’ psychological resources (44). When facing threats of skill depreciation and role marginalization, employees are more likely to experience negative emotions and resource exhaustion (28, 35). Repeated experiences of job insecurity not only cause emotional tension and sleep disturbances in employees but also intensify their pessimistic expectations for future career development due to the persistent perception of health threats (45).

Under the SOR framework, Gen AI is an emerging and disruptive external force. It reshapes job responsibilities and task requirements, forcing employees to update their assessments of job security and triggering feelings of job insecurity (15, 28). Gen AI can handle complex cognitive tasks in knowledge-intensive positions (31, 46). This ability to cross cognitive boundaries heightens employees’ uncertainty about their future career development. As job roles are partially replaced and boundaries are restructured (30, 32, 36), employees who struggle to adapt to technological changes promptly will experience stronger feelings of insecurity (43). Furthermore, individuals’ perceived states also serve as the key mediating mechanism through which environmental stimuli ultimately impact their responses (28). For instance, this “technological oppression” caused by Gen AI not only depletes psychological resources but also weakens employees’ positive acceptance of AI (30). In other words, the job substitution effects and blurring of job roles caused by Gen AI trigger employees’ job insecurity (31, 36), which, in turn, is related to psychological distress. Therefore, we propose the following hypotheses:

*H2a:* Job insecurity is positively associated with employees’ psychological distress.

*H2b:* Job insecurity plays a mediating role between Gen AI use and employees’ psychological distress.

#### 2.2.3 The direct and mediating roles of workplace loneliness (O) on psychological distress (R)

Building and maintaining stable, positive interpersonal relationships is a fundamental motivation for humans (47, 48). The SOR framework suggests that when employees’ emotional needs in interpersonal interactions at the workplace are not met, they may develop negative responses (27, 39, 49). Workplace loneliness depletes employees’ limited cognitive and emotional resources, making it difficult for them to benefit from positive workplace relationships with colleagues, such as the exchange of work-related information (50, 51), and may thereby trigger psychological distress. Moreover, without peer support and effective communication, employees who experience prolonged social loneliness are more prone to negative self-perception and emotional exhaustion, thereby worsening psychological distress such as insomnia, anxiety and depression (32, 49, 52).

Furthermore, workplace loneliness may also play a key mediating role, bridging antecedent stimuli and subsequent responses (27, 28). Although using Gen AI improves task efficiency, it comes at the cost of weakening social interactions (32). Functions of Gen AI, such as text writing and customer response, make employees more dependent on the system when handling tasks, reducing face-to-face collaboration (53). Especially in multitasking contexts, the individual-oriented work style has gradually replaced traditional team collaboration, thereby forming a “social substitution effect,” weakening employees’ connections with others in the workplace, and then leading them to lose social connections and a sense of belonging gradually (32, 54). Work interactions between employees and AI are mechanical and lack human-centered warmth, which can worsen employees’ sense of workplace loneliness (55). Work loneliness drives employees to respond negatively, further exacerbating a destructive cycle. As they tend to judge themselves more adversely, resulting anxiety and insomnia amplify their sense of loneliness, hindering their attempts to develop and maintain meaningful social connections, thus creating a negative feedback loop (55, 56). Based on the above mechanisms, our study proposes the following hypotheses:

*H3a:* Workplace loneliness is positively associated with psychological distress.

*H3b:* Gen AI use is positively associated with psychological distress through the mediating role of workplace loneliness.

#### 2.2.4 The moderating role of information literacy

Additionally, some contextual factors, such as information literacy, may serve as boundary conditions that shape the relationship between Gen AI use and job insecurity. Information literacy is a key individual resource that refers to the ability to identify, evaluate, integrate, and effectively utilize digital information (57, 58). From a job demands–resources perspective, when employees face high-intensity task demands or environmental changes, adequate psychological and support resources can effectively mitigate negative psychological responses and enhance work adaptability (43). As a critical moderating resource in the digital work environment, information literacy not only determines how employees assess technological threats but also plays a “perceptual buffering” role in their psychological adaptation process (46, 58). Individuals with stronger information literacy can more positively interpret changes in the external environment, adjust their adaptation strategies accordingly, and view Gen AI as an extension of their capabilities and a professional tool, thereby reducing insecurity caused by technological uncertainty (46, 57, 59, 60). When facing in-depth AI involvement, they typically possess stronger technical comprehension, problem-solving abilities, and confidence in dealing with new tools, which helps alleviate anxiety brought by technological changes (58, 61). In contrast, employees with lower information literacy, due to a lack of understanding of AI development trends, are more likely to worry about AI replacement and fall into unnecessary anxiety excessively (58, 62). Therefore, we propose the following hypothesis:

*H4:* Information literacy negatively moderates the relationship between Gen AI use and job insecurity.

#### 2.2.5 The moderating role of AI ethical risk perception

AI ethical risk perception is not merely triggered by the frequency of AI use, but stems from individuals’ sensitivity to the ethical uncertainty and ambiguity of AI. While Gen AI enhances intelligent decision-making and interaction capabilities, it also blurs boundaries such as data privacy, algorithmic bias, and responsibility attribution, prompting employees to varying concerns about its ethical consequences (63, 64). The trustworthiness, transparency, and human-like characteristics of AI are key elements shaping individual trust (65, 66). If individuals perceive AI’s decision-making process as opaque or feel a lack of control, it may trigger anxiety and worries about its ethical impacts, thereby affecting their behavioral performance at work (63–65). Perceiving such AI ethical risk may prompt individuals to adopt conservative interaction strategies when facing AI technology, such as reducing communication with colleagues or concealing information related to AI use (67, 68). Especially in workplace contexts involving data privacy and ethical decision-making, when employees perceive that AI use may bring ethical risks, they are more inclined to adopt behavioral inhibition or social withdrawal strategies, avoiding AI-related tasks and discussions (67, 68). Such behaviors, in turn, exacerbate information and social isolation in the workplace, leading employees to experience workplace loneliness. Specifically, when employees perceive that AI use may involve ethical risks, they develop moral anxiety and exhibit social avoidance behaviors, which ultimately intensify workplace loneliness during Gen AI use (32, 63, 64). Based on the above, we propose the following hypothesis:

*H5:* AI ethical risk perception positively moderates the relationship between Gen AI use and workplace loneliness.

**Figure 2:**
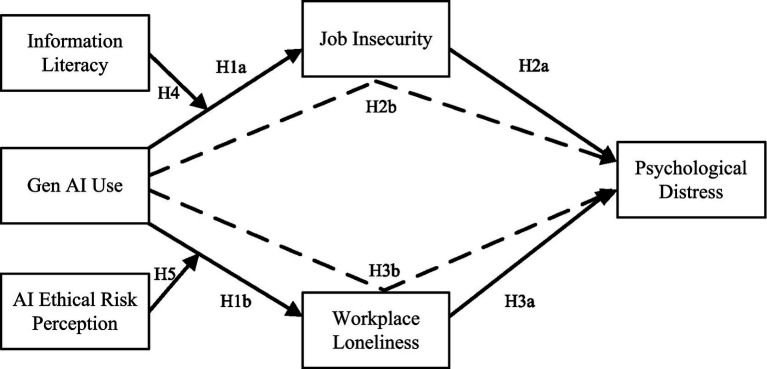
Theoretical model for research hypotheses.

In summary, our research model and hypotheses are illustrated in Figure 2.

## 3 Materials and methods

### 3.1 Variable measurement

All measurements in our study were derived from prior studies with established reliability and validity. The translation/back-translation procedure was used to translate the English items into Chinese. We rated the continuous focal variables by adopting a 5-point Likert scale (from 1 = strongly disagree to 5 = strongly agree) except for psychological distress, which was measured on a 4-point Likert scale. All constructs of variables, item content, squared multiple correlations (SMC), composite reliability (CR), standard factor loadings, and Cronbach’s alpha values are listed in the Supplementary Table 1. Additionally, following the suggestion of Bernerth et al. (69) and Zhang et al. (70), we did not include other control variables in order to keep the model brief and achieve a pure effect.

#### 3.1.1 Psychological distress

We used the General Health Questionnaire (GHQ-12) developed by Goldberg et al. (71) to measure this variable. The GHQ-12 consists of 12 items (6 positive items and 6 negative items) that assess various symptoms of psychological distress, including sleep, tension, anxiety, stress, and happiness. The scale uses a 4-point scale, where 1 represents “never,” and 4 represents “always.” Higher scores indicate higher levels of distress. This scale is considered a standard measure of distress and has been shown to have good applicability in the Chinese context (72). In this study, 8 items (2 positive items and 6 negative items) were ultimately retained after filtering by a factor loading threshold of 0.50. We confirmed that these retained items adequately capture the core constructs of psychological distress, covering key symptoms such as sleep disturbances, anxiety, stress, and loss of self-confidence. The revised scale yielded a Cronbach’s alpha value of 0.867.

#### 3.1.2 Gen AI use

We used the work-related Gen AI use scale developed by Zhang et al. (73). The scale includes four measurement items, such as “In my daily work, I use Gen AI tools to obtain ideas and participate in work-related discussions.”

#### 3.1.3 Job insecurity

We adapted the scale developed by Kim et al. (74), which asks respondents four questions about their feelings of job insecurity caused by artificial intelligence. Based on the original questionnaire, we modified it to suit the theme of this study by incorporating Gen AI scenarios, such as “The introduction of Gen AI technology may make my job insecure.”

#### 3.1.4 Workplace loneliness

We used the scale developed by Wright et al. (37). Wright et al. reviewed the conceptualization and development of workplace loneliness scales and developed a specialized scale. Our study extracted four measurement items, such as “At work, there are very few people with whom I can openly share my personal thoughts.” The Cronbach’s alpha was 0.890.

#### 3.1.5 AI ethical risk perception

Based on the original scale developed by Chiu et al. (46), we modified the items to suit the theme of this study by incorporating Gen AI scenarios. This latent variable includes four measurement items, such as “I am concerned about the risk of non-compliance in the application of results because there are no clear legal provisions regarding Gen AI outputs.”

#### 3.1.6 Information literacy

Lund et al. (75) developed an information literacy scale and investigated its relationship with ChatGPT. We extracted four measurement items from Lund et al.’s scale, such as “I am confident in my ability to assess the credibility and reliability of information sources.” The Cronbach’s alpha was 0.865.

### 3.2 Sample and data collection

The research team contacted 20 enterprises in Kunming, Yunnan Province, China, spanning sectors in the information transmission, software and information technology services, manufacturing, education and research, public services, government departments, and other types of services. The research team, with the assistance of managers, distributed electronic questionnaires generated by the Questionnaire Star platform through internal work groups and other channels. Before the questionnaire items were answered, we explained the research objectives, data anonymity, and ethical commitments to the employees. Ultimately, 461 questionnaires were received. After excluding low-quality questionnaires with short response times or incomplete responses, 424 valid questionnaires were retained, for a response rate of 91.97%. The descriptive statistics of the basic information of the surveyed individuals are presented in Table 1.

**Table 1:** Demographic characteristics of respondents.

Characteristic	Category	Frequency	Percentage (%)
Gender			
Male		123	29.0
Female		301	71.0
Education			
High school or below		136	32.1
Associate degree		117	27.6
Bachelor’s degree		167	39.4
Postgraduate or above		4	0.9
Age			
18–35		71	16.7
36–45		313	73.8
>45		40	9.5
Position			
General staff		327	77.1
Grassroots manager		47	11.1
Middle manager		41	9.7
Senior manager		9	2.1
Organizational size			
≤100		297	70.0
101--300		71	16.8
301--1000		34	8.0
>1000		22	5.2

### 3.3 Test of common method bias

We employed Harman’s single-factor analysis to test the possibility of common method bias (CMB) (76). As recommended by Podsakoff et al. (77), a cumulative variance explanation rate of less than 50% for the single factor is acceptable. The test results of this study showed that the first principal component accounted for 30.498% of the variance, indicating that the first factor was far from explaining the majority of the variance. Thus, it can be preliminarily inferred that the common method bias in the measurement data of this study is minimal and unlikely to significantly bias the results.

## 4 Results

### 4.1 Measurement model analysis

The study tested the quality of the measurement model in terms of reliability, convergent validity, and discriminant validity, with the results presented in Supplementary Table 1 and Table 2. As shown in Supplementary Table 1, all Cronbach’s alpha values were above 0.7, and the composite reliability (CR) values were all above 0.8, indicating that the internal consistency reliability of the questionnaire measurement results was satisfactory. As shown in the third column of Supplementary Table 1, the standardized factor loadings of most items were greater than 0.6, with only three items ranging between 0.5 and 0.6, which is still within the acceptable range. As presented in Table 2, all average variance extracted (AVE) values were higher than 0.45, indicating that the measured variables generally had good convergent validity. Furthermore, the square root of all AVE values was greater than the Pearson correlation coefficients between latent variables, demonstrating good discriminant validity among variables. The above analysis collectively indicates that the measurement model of variables has good reliability, convergent validity, and discriminant validity.

**Table 2:** Tests of convergent validity and discriminant validity of variables.

Latent variable	Mean	Standard deviation	AVE	Discriminant validity					
Gen AI use	3.054	1.064	0.767	**0.876**					
Job insecurity	3.059	1.101	0.687	0.279*	**0.829**				
Workplace loneliness	2.739	1.072	0.671	0.292*	0.519*	**0.819**			
AI ethical risk perception	3.206	1.092	0.745	0.124*	0.683*	0.475*	**0.863**		
Information literacy	3.440	0.931	0.624	0.404*	0.304*	0.168*	0.254*	**0.790**	
Psychological distress	2.217	0.697	0.462	0.087*	0.324*	0.493*	0.271*	−0.07	**0.680**

*p < 0.10. The Pearson correlation matrix is shown on the right side of the table. The square root of the AVE for each construct is shown on the diagonal of the correlation matrix. (the bold values).

### 4.2 Hypothesis testing

After confirming that the measurement model met the requirements, this study constructed a structural equation model based on the aforementioned hypotheses to evaluate and analyze the relational paths. Following the suggestion by Iacobucci et al. (78), the SEM method is more suitable for our research; therefore, we used Mplus 7.4 software to test the structural model containing mediating variables. Regarding the model fit indices, according to the recommendations by Anderson and Gerbing (79), a model fits the actual data well if 1 < χ^2^/df < 3, RMSEA < 0.08, CFI > 0.9, TLI > 0.9, and SRMR < 0.08. The final results of our constructed model were as follows: χ^2^/df = 3.686; RMSEA = 0.080; CFI = 0.917; TLI = 0.905; SRMR = 0.111. Except for the SRMR, other fit indices are very close to the recommended criteria, indicating that the overall level of fit between the model and the data is acceptable.

#### 4.2.1 Tests of direct effects and mediating effects

**Figure 3:**
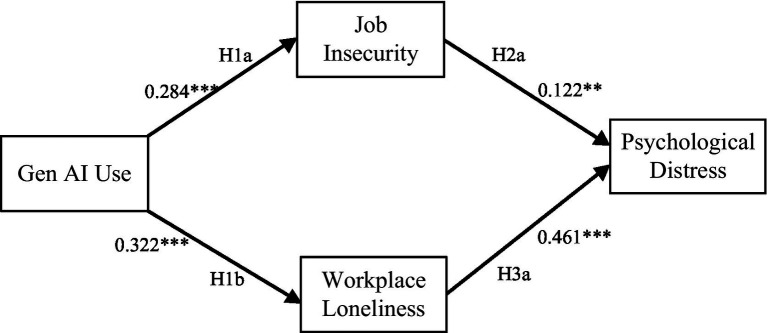
Results of direct path relationship tests between variables. *p < 0.10; **p < 0.05; ***p < 0.01.

As shown in Figure 3, we first tested the hypotheses regarding the direct relationships between variables. Based on the theoretical framework of this study, Gen AI use is positively related to job insecurity (*β* = 0.284, *p* < 0.01), meaning that as employees’ frequency of Gen AI use increases, their perceived job insecurity also rises, thus supporting Hypothesis H1a. Gen AI use is also positively related to workplace loneliness (*β* = 0.322, p < 0.01), so Hypothesis H1b is supported. Additionally, job insecurity is positively correlated with psychological distress (*β* = 0.122, *p* < 0.05), indicating that job insecurity increases employees’ psychological distress, and Hypothesis H2a is supported. Workplace loneliness is also significantly positively correlated with psychological distress (*β* = 0.461, p < 0.01), meaning that the stronger the workplace loneliness, the more severe employees’ psychological distress, thus supporting Hypothesis H3a.

We further conducted tests using the bias-corrected Bootstrap sampling method with 5,000 resamples, as this method provides more stable results (80). Under the bias-corrected 90% confidence interval, the effect is considered significant if the interval does not contain 0. As shown in columns 5–6 of Panel A in Table 3, all direct effects are significant. The mediating effect test results presented in Panel B of Table 3 are also significant. Gen AI use is associated with psychological distress via the mediating role of job insecurity [unstandardized coefficient = 0.020, CI = (0.003, 0.048)], thus supporting Hypothesis H2b. In addition, Gen AI use is also indirectly correlated with psychological distress through the path of workplace loneliness [unstandardized coefficient = 0.087, CI = (0.053, 0.134)], and Hypothesis H3b is verified.

**Table 3:** Test results of direct and indirect relationships between variables.

Influence path	Coef.		S. E.	Bias-corrected 90% CI		Hypothesis verification
	Std. Coef.	Unstd. Coef.		Lower bound	Upper bound	
Panel A: Direct effects						
Gen AI Use → Job insecurity	0.284	0.296	0.049	0.168	0.428	H1a support
Gen AI Use → Workplace loneliness	0.322	0.341	0.048	0.212	0.471	H1b support
Job insecurity → Psychological distress	0.122	0.069	0.058	0.004	0.138	H2a support
Workplace loneliness → Psychological distress	0.461	0.255	0.051	0.179	0.336	H3a support
Panel B: Indirect effects						
Gen AI Use → Job insecurity → Psychological distress	0.020		0.013	0.003	0.048	H2b support
Gen AI use → Workplace loneliness → Psychological distress	0.087		0.024	0.053	0.134	H3b support

Direct effects include standardized coefficients and unstandardized coefficients. Indirect effects are unstandardized coefficients. Confidence intervals are associated with unstandardized coefficients. Bias-corrected 90% CI refers to bias-corrected 90% confidence intervals based on 5,000 bias-corrected bootstrapping resamples.

#### 4.2.2 Test of moderating effects

**Figure 4:**
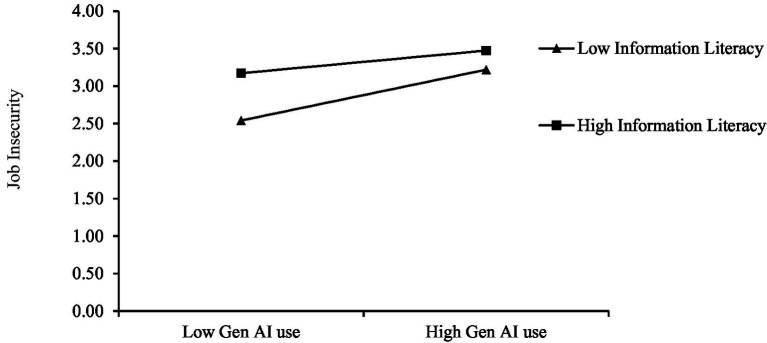
Plot of the moderating effect of information literacy on the relationship between Gen AI use and job insecurity.

We also employed the bias-corrected Bootstrap method based on Mplus 7.4 software to conduct moderation effect tests. As shown in Panel A of Table 4, the cross-term coefficient for the interaction between Gen AI use and information literacy is −0.095, with a confidence interval of [−0.175, −0.012]. This indicates that information literacy has a significant negative moderating effect on the relationship between Gen AI use and job insecurity, i.e., the higher the level of information literacy of employees, the weaker the relationship between Gen AI use and job insecurity. Hypothesis H4 is supported. According to the recommendations of Aiken and West (81), the moderating variables were distinguished by adding or subtracting one standard deviation from the mean value, and an effect diagram was drawn to more clearly present the interaction effect of Gen AI use and moderating variables on the dependent variables. As shown in Figure 4, compared with the low information literacy group, the coefficient of influence of Gen AI use on job insecurity in the high information literacy group decreased from 0.318 to 0.140. This indicates that although the regression coefficient slope is still positive, it has become flatter, demonstrating the negative moderating effect of information literacy. Under conditions where the moderating effect is significant, Panel B of Table 4 further presents the results of the moderated mediation effect test. The results show that different levels of information literacy have a significant moderating effect on the mediating path of “Gen AI use → job insecurity → psychological distress.” The intergroup difference coefficient of the indirect effect between the high and low groups was −0.017, with a confidence interval of [−0.045, −0.002]. In other words, compared to low information literacy, high information literacy can mitigate the indirect relationship between Gen AI use and psychological distress through the mediating pathway of job insecurity.

**Table 4:** Tests of moderating effects and moderated mediating effects of information literacy.

Coefficient conditions	Path	Coef.	S. E.	Bias-corrected 90% CI	
				Lower bound	Upper bound
Panel A: Tests of moderating effects					
Gen AI use * Information literacy	Gen AI use →Job insecurity	−0.095	0.050	−0.175	−0.012
High Information Literacy (Mean + 1 SD)	Gen AI use →Job insecurity	0.140	0.082	0.003	0.273
Low information literacy (Mean - 1 SD)	Gen AI use →Job insecurity	0.318	0.091	0.164	0.463
Panel B: Tests of moderated mediating effects					
High information literacy group (Mean+1SD)	Gen AI use →Job insecurity→Psychological distress	0.013	0.011	0.001	0.038
Low information literacy group (Mean-1SD)	Gen AI use →Job insecurity→Psychological distress	0.030	0.018	0.007	0.066
Difference in indirect effects between high and low groups	Gen AI use →Job insecurity→Psychological distress	−0.017	0.013	−0.045	−0.002

The Coef. is unstandardized coefficient. Bias-corrected 90% CI refers to bias-corrected 90% confidence intervals based on 5,000 bias-corrected bootstrapping resamples.

**Figure 5:**
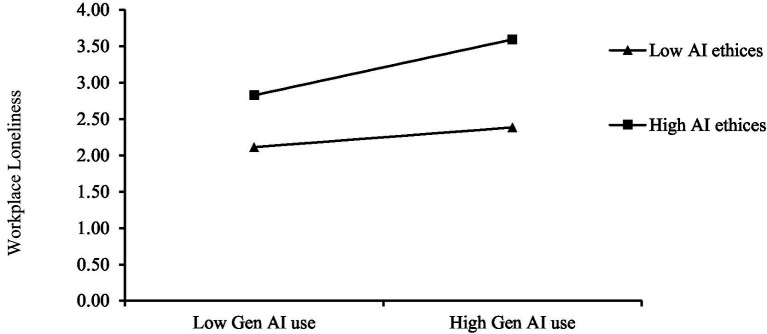
Plot of the moderating effect of AI ethical risk perception on the relationship between Gen AI use and workplace loneliness.

We also examined the moderating role and moderated mediating effect of AI ethical risk perception, as shown in Panel A of Table 5. The cross-term coefficient of Gen AI use and AI ethical risk perception is 0.107, with a confidence interval of [0.048, 0.159]. This indicates that AI ethical risk perception has a significant positive moderating effect on the relationship between Gen AI use and workplace loneliness, i.e., the higher the AI ethical risk perception of employees, the more it enhances the association between Gen AI use and workplace loneliness. The results support Hypothesis H5. Figure 5 shows the positive moderating effect of AI ethical risk perception. Compared with the low AI ethical risk perception group, the coefficient of Gen AI use on workplace loneliness increased from 0.127 to 0.359 in the high AI ethical risk perception group. It indicates that the regression coefficient slope is not only positive but also becomes steeper. In other words, the higher the AI ethical risk perception of employees, the more workplace loneliness is enhanced in the process of using Gen AI. As shown in Panel B of Table 5, different levels of AI ethical risk perception also have a significant moderating impact on the mediating path of “Gen AI use → workplace loneliness → psychological distress.” The indirect effect coefficient between the high and low groups is 0.065, with a confidence interval of [0.030, 0.103]. This suggests that AI ethical risk perception can intensify the relationship between Gen AI use and psychological distress through the mediating path of workplace loneliness.

**Table 5:** Tests of moderating effects and moderated mediating effects of AI ethical risk perception.

Coefficient conditions	Path	Coef.	S. E.	Bias-corrected 90% CI	
				Lower bound	Upper bound
Panel A: Tests of moderating effects					
Gen AI Use * AI ethical risk perception	Gen AI use →Workplace loneliness	0.107	0.034	0.048	0.159
AI ethical risk perception (Mean+1SD)	Gen AI use →Workplace loneliness	0.359	0.077	0.226	0.480
AI ethical risk perception (Mean-1SD)	Gen AI use →Workplace loneliness	0.127	0.050	0.050	0.214
Panel B: Tests of moderated mediating effects					
High AI ethical risk perception group (Mean+1SD)	Gen AI use →Workplace loneliness→Psychological distress	0.100	0.026	0.061	0.146
Low AI ethical risk perception group (Mean-1SD)	Gen AI use →Workplace loneliness→Psychological distress	0.035	0.015	0.014	0.064
Difference in indirect effects between high and low groups	Gen AI use →Workplace loneliness→Psychological distress	0.065	0.023	0.030	0.103

The Coef. is unstandardized coefficient. Bias-corrected 90% CI refers to bias-corrected 90% confidence intervals based on 5,000 bias-corrected bootstrapping resamples.

## 5 Discussion

### 5.1 Theoretical implications

The theoretical contributions of our study include the following aspects. First, our study provides a comprehensive theoretical framework that explains the mechanism through which Gen AI use is correlated with employees’ psychological distress, thereby extending the existing literature on the negative outcomes of Gen AI use. Recently, Sharif et al. found that Gen AI exerts a negative impact on psychological well-being, affecting dimensions such as positive emotions, engagement, interpersonal relationships, meaning, and achievement (7). By validating the relationship between Gen AI and psychological distress, we complement the perspective of Sharif et al. from a different angle: Gen AI use not only impairs psychological well-being but also correlates with a series of manifestations such as anxiety, depression, stress, and pessimism (9). More importantly, we further reveal the dual mediating pathways through which Gen AI use is related to psychological distress. The findings regarding the mediating pathway of job insecurity validate the viewpoints of Li et al. (6) and Sharif et al. (7), who found that this pathway contributes to turnover intention and impaired psychological well-being, respectively. In contrast, we provide additional evidence that psychological distress serves as a negative outcome under this pathway. Our study also identifies a novel mediating role of workplace loneliness, which has largely been overlooked in previous literature. Although prior studies suggest that interactions with AI chat can alleviate loneliness (82, 83), such evidence predominantly pertains to non-work-related AI use in daily life contexts. In contrast, our findings indicate that work-related Gen AI use increases workplace loneliness, which subsequently exacerbates employees’ psychological distress. Additionally, our study supplements the previously identified fragmented evidence regarding the adverse psychological consequences of Gen AI use (13–16). By employing a validated psychological distress scale, we provide empirical support for the relationships among these constructs. More critically, by verifying the underlying mediating mechanisms, our study offers insight into how Gen AI use is associated with psychological distress.

Second, by validating that information literacy serves as a moderating factor, our study advances the literature by identifying a boundary condition in the relationship between Gen AI use and its negative outcomes. Our study demonstrates that employees’ information literacy can mitigate the relationship between Gen AI use and job insecurity, and further alleviate the mediating effect on psychological distress through the job insecurity pathway. Compared with previously identified, immutable intrinsic individual characteristics (18, 19), information literacy is more amenable to acquired learning and self-enhancement. Additionally, our study provides actionable strategies for previously identified organizational-level variables (5, 17). Specifically, it suggests that organizations can support employees by training them in AI-related information literacy. Furthermore, our study offers an alternative explanation for the perspective of He et al. (84), who argued that greater AI knowledge may heighten employees’ perceived risk of unemployment, which may further strengthen the relationship between AI and job insecurity (84). In contrast, our results indicate that employees should not merely focus on the acquisition of AI knowledge; instead, they must develop information literacy—the higher-order capacity to critically engage with AI-generated content and harness it as an extension of their professional capabilities. It can effectively buffer the relationship between Gen AI and job insecurity as well as psychological distress.

Third, our study contributes to the literature on Gen AI use by examining boundary conditions associated with AI ethical risk perception, thereby responding to Bai et al.’s call for investigating the ethical dimensions of employees’ Gen AI use (21). Bai et al. found that employees’ heightened ethical awareness regarding Gen AI use correlates with increased ethical reflection, which in turn undermines service innovation behavior (21). In a similar vein, our findings reveal negative outcomes stemming from employees’ heightened sensitivity to AI ethics. Compared to the findings of Bai et al. on behavioral-level impacts, we provide complementary evidence of AI ethics’ psychological-level effects. Our research also provides empirical evidence for prior qualitative discussions on the ethical implications of Gen AI use. Previous qualitative research has suggested that ethical concerns associated with Gen AI use, such as academic integrity, deepfakes, technological bias, and data privacy, may prevent employees from using Gen AI responsibly, triggering a series of issues (85–87). Our findings validate that heightened sensitivity to AI ethical risk perception strengthens the association between Gen AI use and workplace loneliness as well as psychological distress. Our study illuminates potential outcomes stemming from AI ethical concerns during Gen AI use, thereby providing empirical evidence for calls for AI ethical regulation and governance.

### 5.2 Practical implications

First, given that Gen AI use correlates with employees’ psychological distress through the dual mediating pathways of job insecurity and workplace loneliness, both organizations and employees should take appropriate preventive and intervention measures. From an organizational perspective, organizations should establish a comprehensive Gen AI support system, including clear AI application policies and usage guidelines, to help employees understand the positioning and boundaries of AI technology and reduce job insecurity caused by technological uncertainty. Additionally, organizations should prioritize maintaining positive workplace relationships and team collaboration to avoid overreliance on Gen AI tools, thereby preserving social interaction among employees. From an employee perspective, individuals should proactively adjust their perceptions of Gen AI technology, viewing it as a work-support tool rather than a threat. They should continuously learn and enhance their skills to strengthen their irreplaceability while actively maintaining connections with colleagues to avoid social isolation resulting from excessive reliance on Gen AI.

Second, our findings indicate that information literacy can mitigate the association between Gen AI use and job insecurity as well as psychological distress. Therefore, organizations should focus on investing in digital literacy training programs for their employees. The content of these programs should not only include basic knowledge of AI technology, but more importantly, cultivate employees’ abilities to effectively utilize AI-generated information. Importantly, these training programs should be implemented on an ongoing basis and continuously updated in line with rapid advancements in Gen AI technologies. Employees should also take the initiative to enhance their own information literacy. This entails not only acquiring knowledge about AI and improving proficiency in using AI tools but also cultivating critical thinking skills to assess the accuracy, reliability, and potential limitations of AI-generated content. Ultimately, information literacy should be regarded as a valuable competency and prioritized for long-term investment.

Third, given that AI ethical risk perception exacerbates the negative relationship between Gen AI use and workplace loneliness as well as psychological distress, organizations should establish transparent and accountable AI governance frameworks. Specifically, organizations should develop clear AI ethical guidelines and usage norms that cover key issues, such as data privacy protection, boundaries for AI tool usage, and accountability. Furthermore, organizations need to conduct regular AI ethics training to help employees understand and identify potential ethical risks, while providing guidance and support for handling ethical dilemmas. In addition, organizations should establish open communication channels to encourage employees to discuss and provide feedback on ethical issues related to AI use, thereby avoiding social avoidance behavior due to ethical concerns. Employees should actively participate in organizational AI ethics training to improve their awareness of the potential risks of AI technology and learn to make ethical judgments when using AI. At the same time, when faced with AI ethical dilemmas, employees should actively seek support from the organization rather than choosing to avoid the issue, and should leverage collective wisdom to address complex moral challenges.

### 5.3 Limitations

Although this study contributes to the literature on the relationship between Gen AI use and psychological distress, it has some limitations. First, the study utilizes cross-sectional data collected from Kunming, China. As the data are drawn from a single regional context, the findings may not fully generalize to other contexts with different organizational and cultural environments. Prior research has highlighted that organizational culture plays an important role in shaping employees’ reactions to AI use. For instance, Lingmont et al. (5) suggested that authoritarian organizational cultures may strengthen the relationship between technology-related awareness and perceived job insecurity, as such environments can increase employees’ feelings of powerlessness, thereby reducing employees’ perceived ability to cope with or respond to potential threats. Accordingly, the effects of Gen AI use on employees’ psychological responses in this study may be context-dependent. Future research could adopt longitudinal studies and controlled experiments with samples from different regions (e.g., diverse organizational and cultural contexts) to verify the generalizability of our findings and explore potential heterogeneity.

Second, there may be complex relationships between Gen AI use and psychological distress. The contribution of this study lies in constructing a framework for the influencing mechanism of Gen AI use on psychological distress based on the SOR theory. Future studies could explore other potential mediating channels and boundary conditions. For instance, leadership styles and team climate have recently been confirmed to exert significant impacts on employees’ psychological distress (72, 88), and their mechanisms of action in the context of Gen AI use could be further explored and verified based on our framework. Additionally, beyond Gen AI use, other associated variables, such as leader-member exchange (89), also impact employees’ job insecurity, workplace loneliness, and psychological distress. These control variables may limit our ability to fully rule out other mechanisms. Future studies can examine the stability of Gen AI use by controlling for these related variables and can employ approaches such as SEM-fsQCA to examine differences in effects across various feature configurations.

Finally, several concerns related to model evaluation and validation should be acknowledged. Regarding model fit, although most fit indices met the recommended thresholds, the SRMR value exceeded the optimal cutoff. Therefore, the results of model fit should be interpreted with appropriate caution. In addition, common method bias was assessed in this study using Harman’s single-factor test. While this serves as an acceptable initial check, it has been criticized for its limited sensitivity. Future research is encouraged to employ more robust techniques, such as marker variable approaches or one-factor-constrained CFA-based methods, to further validate the findings.

## 6 Conclusion

Our study contributes to the literature by constructing a comprehensive theoretical framework and empirically examining the mediating paths and boundary conditions of Gen AI use on employees’ psychological distress. The results suggested that Gen AI is associated with psychological distress through a dual mediating pathway of job insecurity and workplace loneliness. Second, employee information literacy negatively moderates the relationship between Gen AI use and job insecurity; that is, high information literacy mitigates the relationship between Gen AI use and job insecurity. The study further reveals that employee information literacy can negatively moderate the indirect relationship via job insecurity; that is, as employee information literacy strengthens, the association between Gen AI use and psychological distress through the mediating path of job insecurity weakens. Third, AI ethical risk perception positively moderates the relationship between Gen AI use and workplace loneliness; that is, the relationship between Gen AI use and workplace loneliness tends to be stronger at higher levels of AI ethical risk perception. In addition, AI ethical risk perception also positively moderates the indirect association via workplace loneliness; that is, as employees’ perception of AI ethical risk increases, the indirect association between Gen AI use and psychological distress through workplace loneliness tends to be stronger.

## Data Availability

The raw data supporting the conclusions of this article will be made available by the authors, without undue reservation.

## References

[1] Chompunuch S Lubart T. AI as a helper: leveraging generative AI tools across common parts of the creative process. J Intelligence. (2025) 13:57. doi: 10.3390/jintelligence13050057, 40422657 PMC12112575

[2] Pathak K Prakash G Samadhiya A Kumar A Luthra S. Impact of gen-AI chatbots on consumer services experiences and behaviors: focusing on the sensation of awe and usage intentions through a cybernetic lens. J Retail Consum Serv. (2025) 82:104120. doi: 10.1016/j.jretconser.2024.104120

[3] Pew Research Center Luona L Kim P. U.S. Workers Are More Worried Than Hopeful about Future AI Use in the Workplace. (2025). Available online at: https://www.pewresearch.org/social-trends/2025/02/25/u-s-workers-are-more-worried-than-hopeful-about-future-ai-use-in-the-workplace/ (Accessed March 24, 2026)

[4] Tencent Research Institute. Annual Survey: Chinese Public’s Views and Usage Behaviors Regarding Generative AI. (2025). Available online at: https://news.qq.com/rain/a/20250924A04BY300 (Accessed March 24, 2026).

[5] Lingmont DN Alexiou A. The contingent effect of job automating technology awareness on perceived job insecurity: exploring the moderating role of organizational culture. Technol Forecast Soc Chang. (2020) 161:120302. doi: 10.1016/j.techfore.2020.120302

[6] Li JJ Bonn MA Ye BH. Hotel employee’s artificial intelligence and robotics awareness and its impact on turnover intention: the moderating roles of perceived organizational support and competitive psychological climate. Tour Manag. (2019) 73:172–81. doi: 10.1016/j.tourman.2019.02.006

[7] Sharif MN Zhang L Asif M Alshdaifat SM Hanaysha JR. Artificial intelligence and employee outcomes: investigating the role of job insecurity and technostress in the hospitality industry. Acta Psychol. (2025) 253:104733. doi: 10.1016/j.actpsy.2025.104733, 39826322

[8] Yi X Kumar S. AI awareness and the breakdown of daily recovery: a spillover pathway to work–family strain. Front Public Health. (2025) 13:1738073. doi: 10.3389/fpubh.2025.1738073 41607879 PMC12835287

[9] Sutin AR Luchetti M Stephan Y Terracciano A. Informant-rated change in personality traits, psychological distress, well-being, and social connection with dementia. Arch Gerontol Geriatr. (2023) 115:105218. doi: 10.1016/j.archger.2023.105218, 37837789 PMC10646812

[10] Baquero A. Hotel employees’ burnout and intention to quit: the role of psychological distress and financial well-being in a moderation mediation model. Behav Sci. (2023) 13:84. doi: 10.3390/bs13020084 36829313 PMC9952249

[11] Obeidat D Lau HKC Mencia-Ledo J Siddiqui S Sarawan A Shi Z . Worker health and well-being in Ontario’s electrical sector: a quantitative study of occupational health outcomes. Front Public Health. (2025) 13:1735294. doi: 10.3389/fpubh.2025.1735294 41602050 PMC12833060

[12] Tu Q Huang C Tu B. Social media utilization, mindfulness practice, and psychological distress of nonprofit workers in China: mediation effects of positive and negative affect. Front Public Health. (2025) 13:1405372. doi: 10.3389/fpubh.2025.1405372, 40241948 PMC11999992

[13] Chuang Y-T Chiang H-L Lin A-P. Insights from the job demands–resources model: AI’S dual impact on employees’ work and life well-being. Int J Inf Manag. (2025) 83:102887. doi: 10.1016/j.ijinfomgt.2025.102887

[14] Alessandro G Dimitri O Cristina B Anna M. The emotional impact of generative AI: negative emotions and perception of threat. Behav Inform Technol. (2025) 44:676–93. doi: 10.1080/0144929X.2024.2333933

[15] Zheng J Zhang T. Association between AI awareness and emotional exhaustion: the serial mediation of job insecurity and work interference with family. Behav Sci. (2025) 15:401. doi: 10.3390/bs15040401, 40282023 PMC12024253

[16] Song X Xu L Peng C Pan S Adibi A Wang X . Enhanced creativity at the cost of increased stress? The impact of generative AI on serious games for creativity stimulation. Behav Inform Technol. (2025):1–25. doi: 10.1080/0144929X.2025.2459267

[17] Lu L Zhao J Chen H. Investigating OTA employees’ double-edged perceptions of ChatGPT: the moderating role of organizational support. Int J Hosp Manag. (2024) 120:103753. doi: 10.1016/j.ijhm.2024.103753

[18] Liu Y Li Y Hu L Zhang Q. How does artificial intelligence usage affect the safety behavior of bus drivers? A double-edged sword study. Transport Res F: Traffic Psychol Behav. (2025) 111:32–44. doi: 10.1016/j.trf.2025.02.026

[19] Zhang L-X Li J-M Wang L-L Mao M-Y Zhang R-X. How does the usage of robots in hotels affect employees’ turnover intention? A double-edged sword study. J Hosp Tour Manag. (2023) 57:74–83. doi: 10.1016/j.jhtm.2023.09.004

[20] Stahl BC Eke D. The ethics of ChatGPT–exploring the ethical issues of an emerging technology. Int J Inf Manag. (2024) 74:102700. doi: 10.1016/j.ijinfomgt.2023.102700

[21] Bai JY Wong IA Huan TCT Okumus F Leong AMW. Ethical perceptions of generative AI use and employee work outcomes: role of moral rumination and AI-supported autonomy. Tour Manag. (2025) 111:105242. doi: 10.1016/j.tourman.2025.105242

[22] Deri E Frank D Vukovic D. Exploring the ethical implications of using generative AI tools in higher education. Inform Basel. (2025) 12:36. doi: 10.3390/informatics12020036

[23] Lin CS Kuo Y-F Wang T-Y. Trust and acceptance of AI caregiving robots: the role of ethics and self-efficacy. Comp Human Behav. (2025) 3:100115. doi: 10.1016/j.chbah.2024.100115

[24] Singh K Chatterjee S Mariani M. Applications of generative AI and future organizational performance: the mediating role of explorative and exploitative innovation and the moderating role of ethical dilemmas and environmental dynamism. Technovation. (2024) 133:103021. doi: 10.1016/j.technovation.2024.103021

[25] Mehrabian A Russell JA. An Approach to Environmental Psychology. Cambridge, MA: The MIT Press (1974).

[26] Xu J Benbasat I Cenfetelli RT. The nature and consequences of trade-off transparency in the context of recommendation agents. MIS Q. (2014) 38:379–406. doi: 10.25300/MISQ/2014/38.2.03

[27] Vieira VA. Stimuli–organism-response framework: a meta-analytic review in the store environment. J Bus Res. (2013) 66:1420–6. doi: 10.1016/j.jbusres.2012.05.009

[28] Duong CD Dao TT Vu TN Ngo TVN Tran QY. Compulsive ChatGPT usage, anxiety, burnout, and sleep disturbance: a serial mediation model based on stimulus-organism-response perspective. Acta Psychol. (2024) 251:104622. doi: 10.1016/j.actpsy.2024.104622, 39647449

[29] Leo WWC Laud G Chou CY. Digital transformation for crisis preparedness: service employees’ perspective. J Serv Mark. (2023) 37:351–70. doi: 10.1108/JSM-07-2021-0249

[30] Chang P-C Zhang W Cai Q Guo H. Does AI-driven technostress promote or hinder employees’ artificial intelligence adoption intention? A moderated mediation model of affective reactions and technical self-efficacy. Psychol Res Behav Manag. (2024) 17:413–27. doi: 10.2147/PRBM.S441444, 38343429 PMC10859089

[31] Brougham D Haar J. Smart technology, artificial intelligence, robotics, and algorithms (STARA): employees’ perceptions of our future workplace. J Manag Organ. (2018) 24:239–57. doi: 10.1017/jmo.2016.55

[32] Tang PM Koopman J Mai KM De Cremer D Zhang JH Reynders P . No person is an island: unpacking the work and after-work consequences of interacting with artificial intelligence. J Appl Psychol. (2023) 108:1766–89. doi: 10.1037/apl0001103, 37307359

[33] Carver CS Scheier MF. Control theory: a useful conceptual framework for personality–social, clinical, and health psychology. Psychol Bull. (1982) 92:111–35. doi: 10.1037/0033-2909.92.1.111, 7134324

[34] Spector PE. Perceived control by employees: a meta-analysis of studies concerning autonomy and participation at work. Hum Relat. (1986) 39:1005–16. doi: 10.1177/001872678603901104

[35] Shoss MK. Job insecurity: an integrative review and agenda for future research. J Manag. (2017) 43:1911–39. doi: 10.1177/0149206317691574

[36] Zhao H Wu P. Artificial Intelligence Job Substitution Risks, Digital Self-efficacy, and Mental Health Among Employees. J Occup Environ Med. (2025) 67:e302–e310. doi: 10.1097/JOM.0000000000003335 39971946

[37] Wright SL Burt CDB Strongman KT. Loneliness in the workplace: construct definition and scale development. N Z J Psychol. (2006) 35:59–68.

[38] Cao X Khan AN Zaigham GH Khan NA. The stimulators of social media fatigue among students: role of moral disengagement. J Educ Comput Res. (2019) 57:1083–107. doi: 10.1177/0735633118781907

[39] Cacioppo JT Cacioppo S. The growing problem of loneliness. Lancet. (2018) 391:426. doi: 10.1016/S0140-6736(18)30142-9, 29407030 PMC6530780

[40] Perlman D Peplau LA. Toward a social psychology of loneliness. Pers Relat. (1981) 3:31–56.

[41] Weiss R. Loneliness: The Experience of Emotional and social Isolation. Cambridge, Mass: The MIT Press (1975).

[42] Wiederhold BK. The rise of AI companions and the quest for authentic connection. Cyberpsychol Behav Soc Netw. (2024) 27:524–6. doi: 10.1089/cyber.2024.0309 38916124

[43] Bakker AB Demerouti E. Job demands–resources theory: taking stock and looking forward. J Occup Health Psychol. (2017) 22:273–85. doi: 10.1037/ocp0000056, 27732008

[44] Burgard SA Brand JE House JS. Perceived job insecurity and worker health in the United States. Soc Sci Med. (2009) 69:777–85. doi: 10.1016/j.socscimed.2009.06.029, 19596166 PMC2757283

[45] Ferrie JE Shipley MJ Marmot MG Martikainen P Stansfeld SA Smith GD. Job insecurity in white-collar workers: toward an explanation of association with health. J Occup Health Psychol. (2001) 6:26–42. doi: 10.1037/1076-8998.6.1.26, 11199254

[46] Chiu Y-T Zhu Y-Q Corbett J. In the hearts and minds of employees: a model of pre-adoptive appraisal toward artificial intelligence in organizations. Int J Inf Manag. (2021) 60:102379. doi: 10.1016/j.ijinfomgt.2021.102379

[47] Tatar B Müceldili B Erdil O. How do employees maintain their well-being during loneliness? The power of organizational nostalgia. Manag Res Rev. (2024) 47:622–42. doi: 10.1108/MRR-12-2022-0842

[48] Yang L Murad M Mirza F Chaudhary NI Saeed M. Shadow of cyber ostracism over remote environment: implication on remote work challenges, virtual work environment and employee mental well-being during a COVID-19 pandemic. Acta Psychol. (2022) 225:103552. doi: 10.1016/j.actpsy.2022.103552, 35255285

[49] Konno Y Nagata M Hino A Tateishi S Tsuji M Ogami A . Association between loneliness and psychological distress: a cross-sectional study among Japanese workers during the COVID-19 pandemic. Prev Med Rep. (2021) 24:101621. doi: 10.1016/j.pmedr.2021.101621, 34722134 PMC8546887

[50] Lam LW Lau DC. Feeling lonely at work: investigating the consequences of unsatisfactory workplace relationships. Int J Hum Resour Manag. (2012) 23:4265–82. doi: 10.1080/09585192.2012.665070

[51] Gilmer DO Magley VJ Dugan AG Namazi S Cherniack MG. Relative importance of incivility and loneliness in occupational health outcomes. Occupational Health Science. (2023) 7:531–55. doi: 10.1007/s41542-023-00145-z, 36789369 PMC9910234

[52] Elahi NS Rasheed MA Shahzad F Arslan A Bajwa SU. Exploration of workplace loneliness and subjective well-being in public higher education institutions: a JD-R model perspective in Pakistan. Public Organiz Rev. (2025) 26:215–235. doi: 10.1007/s11115-025-00910-4

[53] Bankins S Ocampo AC Marrone M Restubog SLD Woo SE. A multilevel review of artificial intelligence in organizations: implications for organizational behavior research and practice. J Organ Behav. (2024) 45:159–82. doi: 10.1002/job.2735

[54] Kraut R Patterson M Lundmark V Kiesler S Mukophadhyay T Scherlis W. Internet paradox: a social technology that reduces social involvement and psychological well-being? Am Psychol. (1998) 53:1017–31. doi: 10.1037/0003-066X.53.9.1017, 9841579

[55] Ozcelik H Barsade SG. No employee an island: workplace loneliness and job performance. Acad Manag J. (2018) 61:2343–66. doi: 10.5465/amj.2015.1066

[56] Guo J Abu Talib M Guo B Ren J Liu J. The mediating role of satisfaction with life and social interaction anxiety in the relationship between loneliness and regulatory emotional self-efficacy. Behav Sci. (2025) 15:392. doi: 10.3390/bs15030392, 40150286 PMC11939567

[57] Pilav-Velić A Černe M Trkman P Wong SI Kadić-Abaz A. Digital or innovative: understanding “digital literacy–practice–innovative work behavior” chain. South East Eur J Econ Busi. (2021) 16:107–19. doi: 10.2478/jeb-2021-0009

[58] Caroline A Coun MJ Gunawan A Stoffers J. A systematic literature review on digital literacy, employability, and innovative work behavior: emphasizing the contextual approaches in HRM research. Front Psychol. (2025) 15:1448555. doi: 10.3389/fpsyg.2024.1448555, 39895978 PMC11783849

[59] Audrin B Audrin C Salamin X. Digital skills at work–conceptual development and empirical validation of a measurement scale. Technol Forecast Soc Chang. (2024) 202:123279. doi: 10.1016/j.techfore.2024.123279

[60] Gao P Gao Y. How does digital leadership foster employee innovative behavior: a cognitive–affective processing system perspective. Behav Sci. (2024) 14:362. doi: 10.3390/bs14050362, 38785853 PMC11117572

[61] Santoso H Abdinagoro SB Arief M. The role of digital literacy in supporting performance through innovative work behavior: the case of Indonesia’s telecommunications industry. Int J Technol. (2019) 10:1558–66. doi: 10.14716/ijtech.v10i8.3432

[62] Lissitsa S Chachashvili-Bolotin S. The effect of digital variables on perceived employability in an ethnic minority and the hegemonic group. Israel Affairs. (2019) 25:1082–104. doi: 10.1080/13537121.2019.1670471

[63] Glikson E Woolley AW. Human trust in artificial intelligence: review of empirical research. Acad Manag Ann. (2020) 14:627–60. doi: 10.5465/annals.2018.0057

[64] Rahman HA. The invisible cage: workers’ reactivity to opaque algorithmic evaluations. Adm Sci Q. (2021) 66:945–88. doi: 10.1177/00018392211010118

[65] Shin D. The effects of explainability and causability on perception, trust, and acceptance: implications for explainable AI. Int J Hum Compr Stud. (2021) 146:102551. doi: 10.1016/j.ijhcs.2020.102551

[66] Yu L Li Y. Artificial intelligence decision-making transparency and employees’ trust: the parallel multiple mediating effect of effectiveness and discomfort. Behavioral Sciences. (2022) 12:127. doi: 10.3390/bs12050127, 35621424 PMC9138134

[67] Faraj S Pachidi S Sayegh K. Working and organizing in the age of the learning algorithm. Inf Organ. (2018) 28:62–70. doi: 10.1016/j.infoandorg.2018.02.005

[68] Jakesch M French M Ma X Hancock JT Naaman M. "AI-mediated communication: how the perception that profile text was written by AI affects trustworthiness". In: Proceedings of the 2019 CHI Conference on Human Factors in Computing Systems (2019). 239:1–13. doi: 10.1145/3290605.3300469

[69] Bernerth JB Cole MS Taylor EC Walker HJ. Control variables in leadership research: a qualitative and quantitative review. J Manag. (2018) 44:131–60. doi: 10.1177/0149206317690586

[70] Zhang Y Li J Liu C-H Shen Y Li G. The effect of novelty on travel intention: the mediating effect of brand equity and travel motivation. Manag Decis. (2021) 59:1271–90. doi: 10.1108/MD-09-2018-1055

[71] Goldberg DP Gater R Sartorius N Ustun TB Piccinelli M Gureje O . The validity of two versions of the GHQ in the WHO study of mental illness in general health care. Psychol Med. (1997) 27:191–7. doi: 10.1017/S0033291796004242, 9122299

[72] Li Y Jia Q. Mitigating psychological distress in the workplace: the role of perceived insider status in leader-follower cognitive style congruence. Acta Psychol. (2024) 250:104505. doi: 10.1016/j.actpsy.2024.104505 39357418

[73] Zhang X Yu P Ma L. How and when generative AI use affects employee incremental and radical creativity: an empirical study in China. Eur J Innov Manag. (2025) 29:702–27. doi: 10.1108/EJIM-04-2024-0466

[74] Kim B-J Kim M-J. How artificial intelligence-induced job insecurity shapes knowledge dynamics: the mitigating role of artificial intelligence self-efficacy. J Innov Knowl. (2024) 9:100590. doi: 10.1016/j.jik.2024.100590

[75] Lund B Agbaji D Teel ZA. Information literacy, data literacy, privacy literacy, and ChatGPT: technology literacies align with perspectives on emerging technology adoption within communities. Hum Technol. (2023) 19:163–77. doi: 10.14254/1795-6889.2023.19-2.2

[76] Harman HH. Modern factor Analysis. Chicago: University of Chicago Press (1976).

[77] Podsakoff PM MacKenzie SB Lee J-Y Podsakoff NP. Common method biases in behavioral research: a critical review of the literature and recommended remedies. J Appl Psychol. (2003) 88:879–903. doi: 10.1037/0021-9010.88.5.879, 14516251

[78] Iacobucci D Saldanha N Deng X. A meditation on mediation: evidence that structural equations models perform better than regressions. J Consum Psychol. (2007) 17:139–53. doi: 10.1016/S1057-7408(07)70020-7

[79] Anderson JC Gerbing DW. Structural equation modeling in practice: a review and recommended two-step approach. Psychol Bull. (1988) 103:411–23. doi: 10.1037/0033-2909.103.3.411

[80] Hayes AF. Introduction to Mediation, Moderation, and Conditional Process Analysis, First Edition: A Regression-Based Approach. New York: The Guilford Press (2013).

[81] Aiken LS West SG Reno RR. Multiple Regression: Testing and Interpreting Interactions. London: Sage (1991).

[82] De Freitas J Oğuz-Uğuralp Z Uğuralp AK Puntoni S. AI companions reduce loneliness. J Consum Res. (2026) 52:1126–48. doi: 10.1093/jcr/ucaf040

[83] Liao J Wei X Sun X Fang X Qiu Y Wan X . Parental and peer phubbing and college students’ gen AI dependency: the mediating roles of loneliness and self-efficacy and the moderating role of perception of gen AI. Curr Psychol. (2025) 44:9151–9164. doi: 10.1007/s12144-025-07748-5

[84] He C Teng R Song J. Linking employees’ challenge-hindrance appraisals toward AI to service performance: the influences of job crafting, job insecurity and AI knowledge. Int J Contemp Hosp Manag. (2023) 36:975–94. doi: 10.1108/IJCHM-07-2022-0848

[85] Yoon S-H Yang S-B Lee S-H. Comprehensive examination of the bright and dark sides of generative AI services: a mixed-methods approach. Electron Commer Res Appl. (2025) 70:101491. doi: 10.1016/j.elerap.2025.101491

[86] Broinowski A Martin FR. Beyond the deepfake problem: benefits, risks and regulation of generative AI screen technologies. Media International Australia. (2024):1–17. doi: 10.1177/1329878X241288034

[87] Farangi MR Nejadghanbar H Hu G. Use of generative AI in research: ethical considerations and emotional experiences. Ethics Behav. (2025) 35:527–43. doi: 10.1080/10508422.2024.2420133

[88] Dong Y Li Y. Leader workaholism and subordinates’ psychological distress: the moderating role of justice climate. Acta Psychol. (2024) 246:104288. doi: 10.1016/j.actpsy.2024.104288, 38678832

[89] Choi Y. The moderating effect of leader member exchange on the relationship between workplace ostracism and psychological distress. Asia Pac J Bus Admin. (2019) 11:146–58. doi: 10.1108/APJBA-11-2018-0205

