# Supplementary material for: The impact of generative AI use on employees’ psychological distress: a moderated mediation model
Source: Front Public Health. 2026 May 20;14:1798423. doi: 10.3389/fpubh.2026.1798423 (PMC13230040; doi:10.3389/fpubh.2026.1798423)
Supplement: Supplementary file 1 [file Table_1.docx]

Supplementary Material

**Supplementary Table 1.** The constructs, item content, Std. factor loadings, composite reliability (CR) values, and Cronbach's α values.

| **Latent variable** | **Item Content** | **Std. Loading** | **S.E.** | **P Value** | **SMC** | **CR** | **Cronbach's α** |
| --- | --- | --- | --- | --- | --- | --- | --- |
| Gen AI Use | I use generative AI in my daily work to get ideas and participate in work-related discussions. | 0.819 | 0.018 | 0.000 | 0.671 | 0.929 | 0.928 |
|  | I use generative AI to find solutions to work problems in my daily work. | 0.928 | 0.010 | 0.000 | 0.861 |  |  |
|  | I use generative AI to ask work-related questions in my daily work. | 0.933 | 0.009 | 0.000 | 0.87 |  |  |
|  | I use generative AI to get work-related knowledge in my daily work. | 0.817 | 0.018 | 0.000 | 0.667 |  |  |
| Job  Insecurity | I think the rise and development of generative AI technology may lead to unemployment. | 0.799 | 0.021 | 0.000 | 0.638 | 0.897 | 0.896 |
|  | The implementation of generative AI may make my job insecure. | 0.890 | 0.016 | 0.000 | 0.792 |  |  |
|  | Even if I wanted to, I'm not completely confident I could keep my job if my employer introduced generative AI technology. | 0.800 | 0.021 | 0.000 | 0.64 |  |  |
|  | If my employer is facing economic problems and wants to introduce generative AI technology, my current job may be replaced or disappear. | 0.822 | 0.020 | 0.000 | 0.676 |  |  |
| Workplace Loneliness | I don't believe my workplace is a place for friendship. | 0.797 | 0.022 | 0.000 | 0.635 | 0.891 | 0.890 |
|  | There are very few people at work that I can open up to and share my personal thoughts with. | 0.818 | 0.020 | 0.000 | 0.669 |  |  |
|  | I feel like I'm emotionally distancing myself from the people I work with. | 0.874 | 0.017 | 0.000 | 0.764 |  |  |
|  | When I'm working, I sometimes feel a sense of emptiness. | 0.785 | 0.022 | 0.000 | 0.616 |  |  |
| AI Ethical Risk  Perception | I am concerned about the risk of violations in the application of results, as there are no clear legal liability provisions for the outcomes produced by the use of generative AI products. | 0.840 | 0.017 | 0.000 | 0.706 | 0.921 | 0.920 |
|  | Generative AI products may violate ethical and behavioral codes, potentially causing harm or loss to me or others. | 0.940 | 0.01 | 0.000 | 0.884 |  |  |
|  | I believe that using generative AI products may expose me to some unknown and uncontrollable ethical risks. | 0.804 | 0.019 | 0.000 | 0.646 |  |  |
|  | Generative AI products, if left unchecked, could cause harm and loss to me and others. | 0.862 | 0.015 | 0.000 | 0.743 |  |  |
| Information Literacy | I can easily find the information I need online. | 0.684 | 0.03 | 0.000 | 0.468 | 0.868 | 0.865 |
|  | I know how to use a variety of online search strategies. | 0.781 | 0.024 | 0.000 | 0.610 |  |  |
|  | Before forming an opinion, I seek answers from multiple sources. | 0.859 | 0.019 | 0.000 | 0.738 |  |  |
|  | I am very confident in my ability to assess the credibility and reliability of information sources. | 0.825 | 0.021 | 0.000 | 0.681 |  |  |
| Psychological Distress | Have you been having trouble sleeping lately due to worries? | 0.535 | 0.037 | 0.000 | 0.286 | 0.869 | 0.867 |
|  | Have you felt recently that you have some difficulties that you cannot overcome? | 0.681 | 0.029 | 0.000 | 0.464 |  |  |
|  | Have you recently felt less confident in yourself? | 0.851 | 0.017 | 0.000 | 0.724 |  |  |
|  | Have you recently felt unhappy or depressed? | 0.892 | 0.014 | 0.000 | 0.796 |  |  |
|  | Have you recently had fleeting thoughts that you are a worthless person? | 0.633 | 0.032 | 0.000 | 0.401 |  |  |
|  | Have you been able to enjoy normal day-to-day activities lately? | 0.513 | 0.039 | 0.000 | 0.263 |  |  |
|  | Overall, would you say you are very happy these days? | 0.549 | 0.037 | 0.000 | 0.301 |  |  |
|  | Have you been feeling stressed lately? | 0.679 | 0.029 | 0.000 | 0.461 |  |  |
